# Structural Properties of Thin ZnO Films Deposited by ALD under O-Rich and Zn-Rich Growth Conditions and Their Relationship with Electrical Parameters

**DOI:** 10.3390/ma14144048

**Published:** 2021-07-20

**Authors:** Sushma Mishra, Ewa Przezdziecka, Wojciech Wozniak, Abinash Adhikari, Rafal Jakiela, Wojciech Paszkowicz, Adrian Sulich, Monika Ozga, Krzysztof Kopalko, Elzbieta Guziewicz

**Affiliations:** Institute of Physics, Polish Academy of Sciences, Al. Lotników 32/46, 02-668 Warsaw, Poland; shmbpj@ifpan.edu.pl (S.M.); eilczuk@ifpan.edu.pl (E.P.); wozniakwojtek@gmail.com (W.W.); adhikari@ifpan.edu.pl (A.A.); jakiela@ifpan.edu.pl (R.J.); paszk@ifpan.edu.pl (W.P.); sulich@ifpan.edu.pl (A.S.); ozga@ifpan.edu.pl (M.O.); kopal@ifpan.edu.pl (K.K.)

**Keywords:** atomic layer deposition, zinc oxide, dislocation density, strain, electrical properties, defect engineering

## Abstract

The structural, optical, and electrical properties of ZnO are intimately intertwined. In the present work, the structural and transport properties of 100 nm thick polycrystalline ZnO films obtained by atomic layer deposition (ALD) at a growth temperature (T_g_) of 100–300 °C were investigated. The electrical properties of the films showed a dependence on the substrate (*a*-Al_2_O_3_ or Si (100)) and a high sensitivity to T_g_, related to the deviation of the film stoichiometry as demonstrated by the RT-Hall effect. The average crystallite size increased from 20–30 nm for as grown samples to 80–100 nm after rapid thermal annealing, which affects carrier scattering. The ZnO layers deposited on silicon showed lower strain and dislocation density than on sapphire at the same T_g_. The calculated half crystallite size (D/2) was higher than the Debye length (L_D_) for all as grown and annealed ZnO films, except for annealed ZnO/Si films grown within the ALD window (100–200 °C), indicating different homogeneity of charge carrier distribution for annealed ZnO/Si and ZnO/*a*-Al_2_O_3_ layers. For as grown films the hydrogen impurity concentration detected via secondary ion mass spectrometry (SIMS) was 10^21^ cm^−3^ and was decreased by two orders of magnitude after annealing, accompanied by a decrease in Urbach energy in the ZnO/*a*-Al_2_O_3_ layers.

## 1. Introduction

Extensive research efforts are being made worldwide to overcome the obstacles of conductivity control and its conversion towards p-type in wide bandgap semiconductors, a particular case of which is ZnO. The success of these efforts will ensure the development of practical technologies (e.g., piezo-phototronics, ZnO-based p-n homojunction, UV detectors, thin film transistors) that fully exploit the electronic and optoelectronic properties of this compound [1]. However, the controllability and reproducibility of electronic transport in ZnO films pose a major challenge because the carrier concentration in ZnO films deposited by different growth methods can assume extremely different values, ranging from 10^15^ to 10^21^ cm^−3^. As charged native point defects are believed to be either deep or have high formation energy [2,3,4,5,6], they cannot provide abundant carriers at room temperature (RT). For this reason, high electron concentrations commonly observed in undoped ZnO have been attributed initially to hydrogen impurity introduced unintentionally during the growth process [6,7,8]. Current knowledge suggests that the role of hydrogen is more nuanced. There is strong evidence that interstitial hydrogen, H_i_, plays a role of donor [6,8], while hydrogen molecule, H_2_, has been shown to be electrically inert in ZnO [9,10]. However, recent investigations strongly suggest that hydrogen impurity in ZnO material may be involved in a number of complexes with native point defects, such as V_Zn_·nH, Zn_i_·V_O_·H, and others [2,3,4,5,11,12,13,14]. Some of these complexes introduce shallow donor and acceptor levels that affect the resulting ZnO conductivity [4,10,15,16,17,18].

As several theoretical calculations show, the formation energy of native point defects is considerably affected by the O/Zn growth conditions [2,6,7,19]. Therefore, defects such as oxygen vacancy (V_O_) or zinc interstitial (Zn_i_) have lower formation energy under Zn-rich conditions [20], while zinc vacancy (V_Zn_) or oxygen interstitial (O_i_) have lower formation energy, so they are more abundant under O-rich conditions. Accordingly, it can be expected that such defect complexes as n·V_Zn_, V_Zn_·nH, or V_Zn_·N_O_ are readily formed under O-rich conditions, while Zn-rich conditions favor the formation of such complexes as Zn_i_·V_O_·H, Zn_i_·N_O_, or nZn·V_O_ [6,21,22,23].

Our previous studies performed on 1 μm thick ZnO films grown by atomic layer deposition (ALD) confirmed that donor and acceptor states in these films are affected by film stoichiometry, leading to a significant difference in conductivity [21]. The above study was based on temperature-dependent photoluminescence (PL), where sharp PL lines characteristic of thick films were used to determine the localization and hence binding energy of donors and acceptors. On the other hand, much thinner films (below 200 nm) are of great interest as they are used in many technological fields such as thin film transistors (TFTs) [24] and optoelectronic devices such as LEDs and laser diodes. The study of thinner films is more challenging because, in this case, interface-induced disorder and stress/strain effects cannot be neglected [25]. Moreover, it has been shown that changing the thickness of ZnO films can affect the electrical, structural, and optical properties, which is manifested in electrical conductivity, crystallite size, and the optical band gap [21,22,23,25,26,27].

In the present work, the structural properties, electrical parameters, and concentration of impurities of thin (100–150 nm) ZnO films deposited by ALD at growth temperature (T_g_) of 100 to 300 °C were studied. It has been shown [28,29] that this growth temperature range influences the stoichiometry of the film, changing it from O-rich to Zn-rich as the T_g_ increases from 100 to 200 °C and above. On the other hand, it might be expected that structural defects also play a role by inducing poorly explored dislocation-native defect complexes: dislons that affect the conductivity of the film. In order to explore this point, we have investigated two series of ZnO samples, deposited on Si (100) and on *a*-Al_2_O_3_. The results of the present study are compared with the optical data obtained for these layers [30]. The calculated strain in these films, as well as its variation with T_g_ and correlation with Urbach energy (E_u_), are also discussed.

It should be noted that different methods can be used for the growth of ZnO, such as CVD [31], MOCVD [32], and MBE [33] for epitaxial layers. However, ZnO films grown by the ALD technique are of particular interest as they combine several advantages such as high conformity, large area uniformity, absence of pin holes, and precise thickness control with requirements for industrial applications in terms of possibility of economic growth on large substrates.

The investigations aimed to control the conductivity of ZnO-ALD thin films through native and structural defects and their complexes. The former can be tuned via the stoichiometry of the films by changing it from O-rich to Zn-rich [6,21,22]. Such an approach can be considered as a kind of defect engineering in this material.

## 2. Growth Details and Used Experimental Techniques

Thin ZnO films were deposited by the ALD technique on high resistivity (*ρ* ≅ 5000 ohm cm) Si (100) and *a*-oriented Al_2_O_3_ substrates in a Savannah-100 Cambridge Nanotech reactor using a double-exchange chemical reaction between de-ionized water and diethylzinc [(C_2_H_5_)_2_Zn]. The ALD processes were performed in 1000 cycles with 20 ms pulse time for both precursors, while purging time (N_2_) for deionized water and DEZn was 20 s and 8 s, respectively. It should be noted that the ZnO films were deposited on both substrates together during the same ALD process. The series of samples were obtained at temperatures, T_g_, of 100, 130, 160, 200, 250, and 300 °C. It was shown that, in this T_g_ range, the stoichiometry of the deposited ZnO films changes from O-rich (at 100 °C) to Zn-rich (at 200 °C and above) [6,18]. The thickness of the ZnO/Si layers (100–150 nm) was measured with a reflectometer using a NanoCalc 2000 (Mikropack GmbH, Ostfildern, Germany), and the thickness of the ZnO/*a-*Al_2_O_3_ films (100 nm) was measured with a profilometer (Dektak 6M stylus, Veeco, Tucson, AZ, USA). Structural X-ray diffraction (XRD) measurements were performed with Cu Kα1 radiation (λ = 1.5406 Å) using a Bragg–Brentano PANalytical Empyrean powder diffractometer (PANanalitical, Westborough, MA, USA) with sample spinning. The concentrations of H, C, and N in ZnO films were determined by secondary ion mass spectrometry (SIMS) using a IMS 6f microanalyzer (CAMECA, Gennevilliers Cedex, France). Hall effect measurements were performed on the square (1 × 1 cm^2^) samples in the van der Pauw configuration using an RH2035 PhysTech88 system equipped with a 0.4 T permanent magnet (PhysTech GmbH, Moosburg, Germany). Ti/Au films for the ohmic contacts were deposited using a PVD75 e-beam evaporation system from Kurt Lesker (Jefferson Hills, PA, USA). Annealing processes were performed at 800 °C in an oxygen atmosphere for 3 min using a rapid thermal annealing (RTP) system AccuThermo AW610 from Allwin21 Co. (Morgan Hill, CA, USA). A Carry 5000 UV/vis/NIR spectrophotometer from Agilent Technologies (Blacksburg, Santa Clara, CA, USA) with a PbS detector was used for the absorption and Urbach energy measurements.

A Dimension Icon atomic force microscope (AFM Bruker, Santa Barbara, CA, USA) was used to measure surface roughness in the peak force tapping mode with a ScanAsyst-AIR (Bruker) probe (tip radius of 2 nm). Images of 1 µm × 1 µm and 10 µm × 10 µm were acquired under ambient conditions with a resolution of 512 × 512 measurement points.

## 3. Experimental Results and Discussion

### 3.1. XRD: Film Texture/Preferred Orientation and Dislocation Density

Si(100) and a-oriented Al_2_O_3_ substrates were chosen for a systematic and comparative study to investigate the structural properties of thin ZnO films and relate them with those previously reported for thicker ALD-ZnO/Al_2_O_3_ films [21]. In general, polycrystalline ALD-ZnO films, when deposited on different substrates, have a strong tendency to grow with the polar c-direction, while other preferred orientations are rarely reported for specific substrates and deposition conditions [21,34,35,36,37]. For textured samples, diffractograms collected in the Bragg–Brentano mode provide basic, information on the preferred orientation of crystallites (but not on the distribution of crystallite orientation). The diffraction effects are observed for those crystallites where the direction of the low Miller indices does not deviate more than 1–2 degrees from the normal to the surface. Crystallites that do not meet these conditions do not contribute to diffraction peaks. The preferred orientation refers to the most common direction; usually, the preferred orientation is obtained by analyzing the relative intensities I/I_rand_ value (I_rand_ is the intensity in the reference powder pattern).

**Figure 1:**
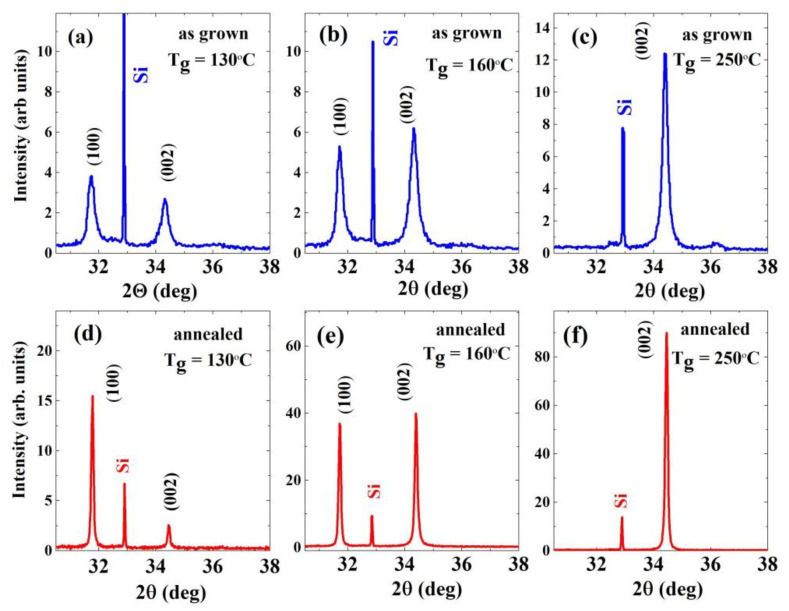
X-ray diffractograms of as grown (**a**–**c**) and annealed (**d**–**f**) ZnO/Si (100) films grown at 100 °C, 160 °C, and 250 °C.

**Figure 2:**
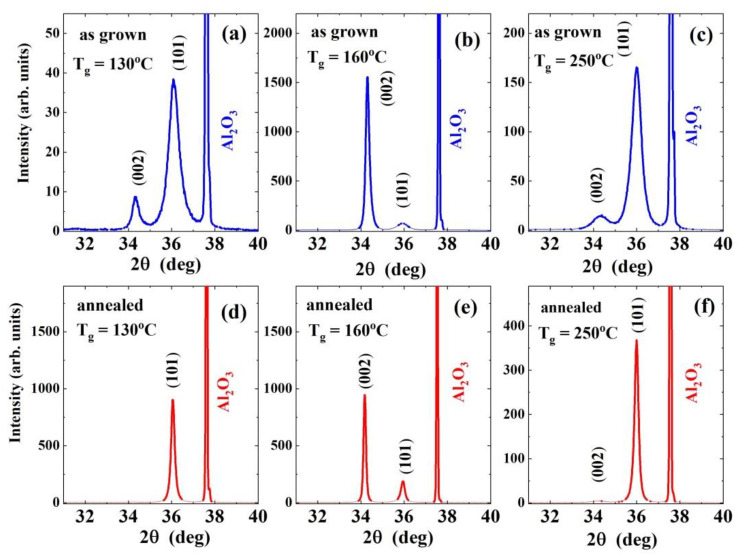
X-ray diffractograms of as grown (**a**–**c**) and annealed (**d**–**f**) ZnO/a-Al_2_O_3_ films grown at 100 °C, 160 °C, and 250 °C.

Diffraction patterns of all investigated films, both as grown and annealed (Figure 1 and Figure 2, Figures S1 and S2 in Supplementary Data) confirm that the wurtzite (w) structured polycrystalline ZnO films are formed at each T_g_, but with different preferred orientation of the crystallites. No additional diffraction peaks corresponding to Zn or other phases are detected.

Despite the fact that the parameters of the crystal lattice do not match, the diffraction patterns show a correlation between the layer orientation and the type of substrate. Two dominant orientations, [100] and [001], are observed for ALD-ZnO/Si(100), and only one preferred orientation, [101], in the case of ALD-ZnO/*a*-Al_2_O_3_, except for T_g_ = 160 °C, where the [001] orientation occurs solely.

As grown ZnO/Si(100) films show ZnO reflections at 31.7° and 34.4°, which are related to the [100] and [001] orientations, respectively. At low T_g_ (100 and 130 °C, Figure 1a), most of the crystallites grow along the [100] direction and peak intensity from the [001] oriented crystallites is lower. However, owing to different growth rates as a function of temperature (higher for the [001] oriented crystallites as compared with the [100] crystallites [38]), the [001] oriented crystallites assume dominance over the [100] crystallites at a temperature of 160 °C (Figure 1b), and at T_g_ = 250 and 300 °C, only the [001] orientation is observed (Figure 1c). In other words, the ZnO/Si films show a switch from the [100] to the [001] orientation at a growth temperature between 160 °C and 200 °C (Figure S1c,f in Supplementary Data).

The switch of crystallite orientation is accompanied by a significant decrease in the intensity of all diffraction peaks. While for the growth temperature range between 100 and 160 °C, the intensity of diffraction peaks increases with T_g_, for the switching temperature of 200 °C, we observe a drop followed by a jump in intensity at the highest T_g_ (see Figure S1 in Supplemental Material).

These results are in line with literature data reporting switching behaviour between [100] and [001] orientations at the switching temperature, T_sw_, ranging from 155 °C to 220 °C [34,38]. Similar to these previous studies, in the present work, we also found three zones based on the dominance of [100] or [001] orientations: (a) Zone 1: 100–160 °C, where both [100] and [001] oriented crystallites were present in the films; (b) Zone 2: 160–200 °C, where a switch in crystallite orientation to [001] dominance was observed; and (c) Zone 3: 250–300 °C, where the [001] direction dominates.

The switching phenomenon can be understood considering the varying oxygen content in the growing film; the O content decreases with T_g_ [27]. It can be supposed that the growth along the preferred orientations in ZnO/Si films depends on the O/Zn ratio. At 100 °C, i.e., when the ZnO films are O-rich, the [100]-oriented crystallites with c-axis parallel to the substrate generally grow, while at temperature above 160 °C, when the films are Zn-rich, the crystallites with c-axis perpendicular to the substrate dominate [21,34,37,38]. The phenomenon of orientation switching has been reported by Pung et al. and assigned to the premature dissociation of DEZn that could occur in the temperature range of 155–220 °C [38].

Premature dissociation of DEZn could probably have occurred in the temperature range of 155–220 °C. In this process, the dissociated ethyl group could be further broken down into ethyl group fragments such as $CH_{3}CH_{2}^{-}$ and $CH_{3}^{-}$. These anions were able to adhere to the positively charged Zn-[001] surface. As a result, the c-axis growth direction was suppressed, forcing the crystals to grow in the [100] orientation. In the high deposition temperature range (Zone 3), these anions could be further decomposed into CO, CO_2_, and H_2_O or desorbed from the substrate surface [30]. Thus, suppression of ZnO crystal growth in the [001] orientation was no longer possible. Therefore, thin ZnO film exhibited a high [001] preferred orientation with enhancement of the [001] peak in Zone 3. However, it should be remembered that the dissociation temperature of the precursor is sensitive to such parameters as chamber pressure and so on, which explains the relatively higher range of switching temperature in our films. It is also worth noting that the ZnO crystal structure has a maximum growth rate along the [001] direction at T_g_ ≅ 160 °C, as it falls within the ALD window. Hence, as has been found earlier [34], the preferential growth with the *c*-axis perpendicular to the surface is associated with a higher growth rate of ZnO films.

In the case of thin as grown ZnO/*a*-Al_2_O_3_ films, two reflections at 36.5° and 34.4° were present, which is consistent with the [101] and [001] orientations of the crystallites, respectively (Figure 2a–c and Figure S2a–f in Supplemental Material). At the lowest T_g_ (100–130 °C), most of crystallites grow with the [101] orientation, while the amount of [001]-oriented crystallites is relatively small (Figure 2a and Figure S2a,b in Supplemental Material). At a growth temperature of 160 °C, we observe an abrupt switch from the [101] orientation to the [001] one, similar to what was observed in the case of the Si(100) substrate.

Surprisingly, at higher growth temperatures (i.e., zone 3, T > 200 °C), only a low intensity of [001] orientation was observed, while the [101]-oriented crystallites dominate the XRD spectra, as happens in the case of the low growth temperature region. The different evolution of the preferred orientation of ZnO/Si(100) and ZnO/a-Al_2_O_3_ films with T_g_ is a fingerprint of the interaction between the growing ZnO layer and the substrate, which exists despite the lack of lattice matching. In such a situation, the growth rate of a particular orientation is anisotropically affected by the presence of strain between the thin ZnO film and the substrate interface [36]. The orientation of the sapphire substrate with an axis perpendicular to the surface restricts the [101] orientation of crystallites in thin ZnO films. It should be noted that, in the case of 900 nm thick ZnO/Al_2_O_3_ films we investigated previously, where weaker interaction with the substrate is expected, four crystallographic orientations ([100], [101], [110], and [001]) have been observed for the 100–200 °C T_g_ range [21].

The strain analysis, further presented in this subsection, reveals tensile strain for both ZnO/Si and ZnO/*a*-Al_2_O_3_ layers. However, the strain evolution with T_g_ was different for both substrates, which could be responsible for the different preferred orientation of the ZnO deposited on these two substrates in the high T_g_ region.

No additional phases were observed after post-growth RTP compared with the as grown films (Figure 1 and Figure 2), but for both substrates, the intensity of the XRD signals increased significantly over the entire T_g_ range (100–300 °C) compared with as grown samples, while the FWHM value of most diffraction peaks decreased considerably, indicating an increase in crystallites’ size.

#### 3.1.1. AFM: Surface Morphology

**Figure 3:**
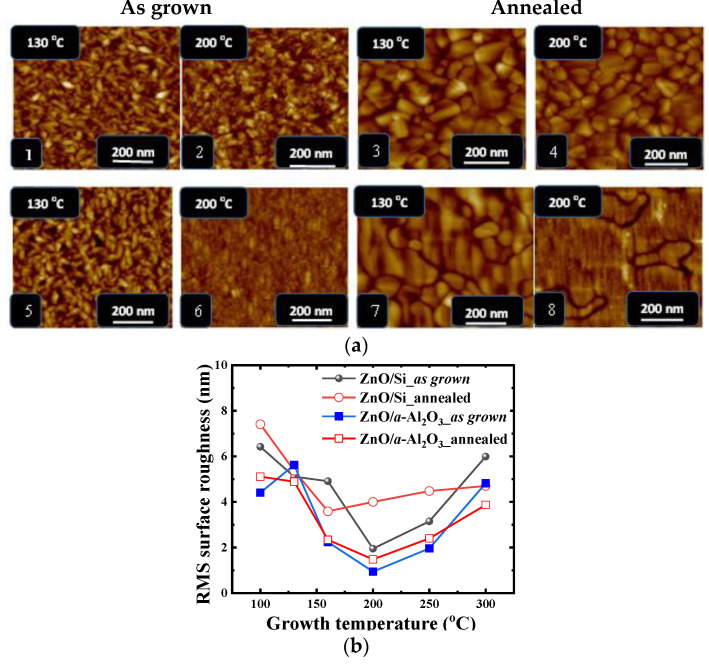
(**a**) Atomic force microscope (AFM) images of as grown (1,2) and annealed (3,4) thin ALD ZnO/Si films and as grown (5,6) and annealed (7,8) ZnO/*a*-Al_2_O_3_ films grown at 130 °C and 200 °C, respectively; (**b**) the graph of roughness variation with T_g_ for all types of as grown/annealed films is also shown below the images.

The surface morphology of the oxide films was found to be strongly dependent on crystallographic orientation and crystallite size (Figure 3). The maximum root mean square (RMS) value of the surface roughness was found for the films grown at T_g_ ≅ 100 °C–130 °C (5.4 nm ZnO/Si and 5.6 nm for ZnO/*a*-Al_2_O_3_). The RMS value gradually decreases with T_g_ to the minimum value of 2 nm for Si (100) and 0.9 nm for Al_2_O_3_ substrate observed at 200 °C, which is consistent with the previous AFM result for ZnO/Si films deposited by ALD [34].

After post growth annealing, an increase in surface roughness was observed for films grown at T_g_ of 100, 200, and 250 °C, but a decrease for films grown at T_g_ of 130, 160, and 300 °C for both ZnO/Si and ZnO/a-Al_2_O_3_ films. However, the films grown in the temperature range above 200 °C showed higher roughness, which further increased with T_g_ to the values observed for the lowest temperature range (Figure 3). It is worth noting that, for all films, the lowest RMS values are observed at 160–200 °C, when switching of crystallographic orientation occurs. It was found that the intensity of the X-ray diffraction peaks is extremely low at the switching temperature, indicating a significant contribution of the amorphous phase. This result is consistent with previous reports on high-k oxides deposited by ALD, which are intentionally deposited amorphous to achieve smoothness at the atomic scale [39].

#### 3.1.2. Crystallite Size

**Figure 4:**
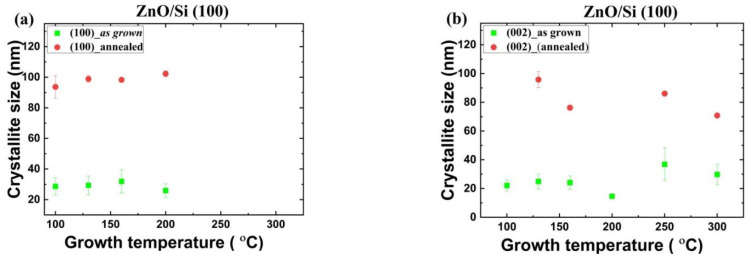
The size of crystallites oriented along (**a**) the [100] and (**b**) [001] direction for ZnO/Si(100) films.

**Figure 5:**
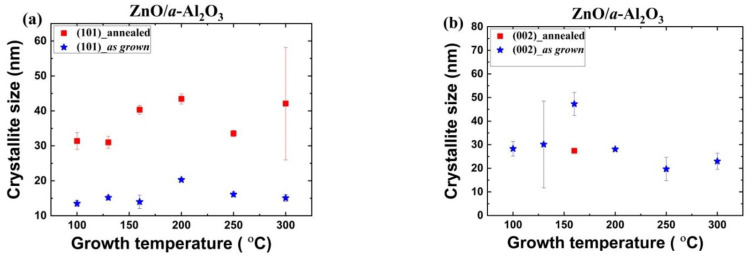
The size of crystallites oriented along (**a**) the [101] and (**b**) [001] direction for ZnO/a-Al_2_O_3_ films.

Analysis of the XRD data using the Scherrer model [40,41,42] reveals that the average crystallite size for as grown films increases from 15 nm to 30–40 nm for ZnO/*a*-Al_2_O_3_ and from 20 nm to 30 nm for ZnO/Si. In detail, the crystallites size for the as grown ZnO/Si samples is 15–37 nm and 26–32 nm for [001]- and [100]-oriented crystallites, respectively, as a function of T_g_ (Figure 4), while for the ZnO/*a*-Al_2_O_3_ samples, the grain size varies between 14 and 20 nm, and 20 nm and 48 nm for [101]- and [001]-oriented crystallites, respectively, as a function of T_g_ (Figure 5).

Subsequently, the rapid thermal annealing (RTP) process was performed in oxygen atmosphere at 800 °C for 3 min (see Figure 4 and Figure 5). After the RTP process, the crystallite size in ZnO/Si films increases to 100 nm and 80 nm for the [100]- and the [001]-oriented crystallites, respectively. For annealed ZnO/*a*-Al_2_O_3_ films, the crystallite size increases to 31–43 nm for the [101]-oriented crystallites and decreases to 25 nm for the [001]-oriented crystallites (Figure 5). This indicates the effect of annealing is more pronounced in the ZnO/Si (100) films than in the ZnO/*a*-Al_2_O_3_ films. At the same time, the intensity of the [002] peak decreases considerably in the ZnO/*a*-Al_2_O_3_ films, so that mainly the [101]-oriented crystallites are observed in the annealed sapphire samples (see Figure S2g–h, in the Supplementary Material).

In this way, the ZnO/*a*-Al_2_O_3_ films showing only the [101]-oriented crystallites were obtained (except T_g_ = 160 °C, where only [002] peak appears). To the best of our knowledge, this is itself an unprecedented report on the thermally stable ZnO thin films showing only [101]-oriented crystallites, because the [001] orientation is usually reported for such films [43].

#### 3.1.3. Structural Defects and Dislocation Density

The crystallite size, investigated in the previous paragraph, is commonly used to evaluate the dislocation density (*δ*), which is an important parameter describing the structural quality of single crystalline solids. It was initially involved based on the XRD microbeam studies of cold-worked metals [44] and more recently applied for polycrystalline ZnO films as well [45]. Evidence from the micro-beam experiments indicated that the metal is broken into blocks, with dislocations located at the boundaries between two adjacent blocks. Under these assumptions, the dislocation density is evaluated by the formula: $\delta =\frac{n}{D^{2}}$*,* where *n* is equal to 1 for isotropic distribution of dislocations, while D is the dimension of the block [46]. The above formula was applied for nonmetallic single crystalline solids and epitaxial films, where D is considered as the crystallite size. For a polycrystalline material, the assumptions of the model are generally not met, and the calculated value $\delta =\frac{1}{D^{2}}$ cannot be treated strictly as the dislocation density; however, the above formula has also been used in this case [46]. In fact, the *δ* value depends on the crystallite size, thus *δ* can be treated as a parameter describing the amount of structural defects and the structural quality of the film, providing a convenient tool for comparison between different layers. Following this interpretation, we determined *δ* for all ZnO films studied and treated the obtained *δ* values with the above-mentioned reservations.

In the case of ZnO films, the evaluation of polycrystalline film quality based on *δ* creates an interesting criterion because grain boundaries and dislocations affect the optical and electrical properties, as some native defects such as Zn vacancies can accumulate near grain boundaries and dislocation cores [47], and the interaction of point defects with structural defects may lead to the formation of “point defect–dislocation complexes” [48,49] that are responsible for certain localized energy levels in this material and play a role of non-radiative recombination centres.

In the investigated ZnO films, one or two reflections appeared with relative intensity and FWHM, depending on the substrate and T_g_. Accordingly, one or two differently oriented crystallite types were observed in the films, each with a specific intensity and crystallite size. In many cases, the intensity of two diffraction peaks was comparable (see Figure S1a–d), so the preferred orientation could not be indicated.

In order to account for this diversity, we calculated the $\delta =\frac{1}{D^{2}}$ value for each orientation separately and then calculated the weighted average *δ*_avg_ value for all of the films. For the ZnO/Si (100) films (Figure 1a–c), the *δ*_avg_ was calculated as follows:

$$\delta_{avg}=\delta_{100}\frac{I_{100}^{*}}{I_{100}^{0}}+\delta_{002}\frac{I_{002}^{*}}{I_{002}^{0}}$$

where $\delta_{100}=\frac{1}{D_{100}^{2}}$, $\delta_{002}=\frac{1}{D_{002}^{2}}$, $I_{100}^{*}=\frac{I_{100}}{I_{100}+I_{002}}$, and $I_{002}^{*}=\frac{I_{002}}{I_{100}+I_{002}}$, while $I_{100}^{0}$ and $I_{002}^{0}$ are relative intensities listed in the JCPDS data file [file No. 36-1451]. For the ZnO/Al_2_O_3_ films, the same formula was used with corresponding parameters of the [101] and [002] peaks observed in these films (see Figure 2). The average dislocation densities calculated according to the above procedure are given in Table 1 and Table 2. For as grown films deposited on both substrates, an *δ*_avg_ value of 10^11^ lines/cm^2^ was found, which is similar to the values previously obtained for polycrystalline ZnO films [45,50], and ZnO/Al_2_O_3_ epilayers [51].

However, the magnitude of *δ*_avg_ is very different for both substrates and is 2–3 times lower for the Si substrate compared with sapphire for each T_g_, except the temperature range of 160–200 °C, when a switch of crystallographic orientation is observed. After RTP, the *δ*_avg_ value drops for both substrates, but this effect is much more pronounced for the Si(100) because, in this case, the decrease of more than one order of magnitude is observed, leading to a dislocation density of about 10^10^ lines/cm^2^. For the annealed ZnO/*a*-Al_2_O_3_ films, the *δ*_avg_ value drops about threefold and, in most cases, is about 5 to 10 times higher than for Si substrate (sees Table 1 and Table 2).

It is worth noting that significant changes in the average dislocation density were observed near the switching temperature. For the Si (100) substrate, a threefold increase in *δ*_avg_ value followed by an abrupt decrease appeared at T_g_ ≅ 200 °C, and was seen after the RTP process as well. For the *a*-Al_2_O_3_ substrate, an increase followed by an abrupt decrease in the *δ*_avg_ value was seen at T_g_ 160 °C and 200 °C, as a result of the aberration of the dislocations coming from the switching of the preferred orientation.

**Figure 6:**
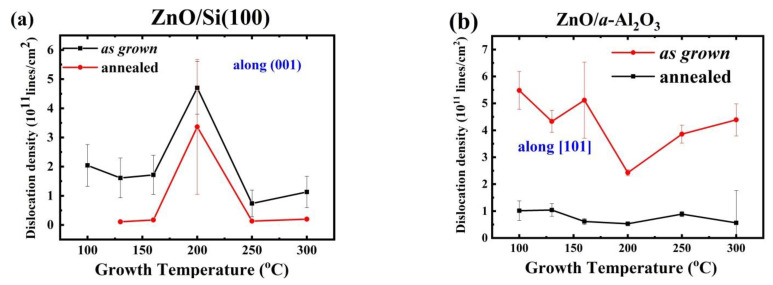
Dislocation density versus T_g_ for as grown and annealed films (**a**) along the preferentially oriented [001] crystallites in ZnO/Si films and (**b**) along the [101]-oriented crystallites in ZnO/*a*-Al_2_O_3_ films.

To visualize this effect, the dislocation density *δ* is plotted versus T_g_ with respect to the preferred orientations, [001] for Si (100) and [101] for *a*-Al_2_O_3_ (Figure 6a,b).

It can be expected that the amount of grain boundaries, expressed by *δ*_avg_, influences the concentration of structural defects and defect complexes occurring in ZnO/Si(100) and ZnO/*a*-Al_2_O_3_ films, and thus may be the origin of the observed conductivity/carrier concentration differences in the investigated films. This issue will be discussed in more detail in Section 3.3.

#### 3.1.4. Strain Analysis

The presence of different crystallographic orientations depending on the type of substrate and the deposition temperature prompts us to investigate a role of strain in the ZnO layer, which is expected to affect the electrical and optical properties. Naturally, there is both an extrinsic and intrinsic type of strain coexisting in ZnO films. Crystallographic imperfections in the ZnO crystal lattice caused by a high density of (i) hydrogen and hydrogen-related complexes, (ii) oxygen vacancies (V_O_) (iii) zinc interstitials (Zn_i_), (iv) zinc vacancies, (v) various types of dislocations, and (vi) grain boundaries (GBs) could be responsible for the intrinsic strain [5,7,47].

A large mismatch in lattice constants and differences in thermal expansion coefficients between the ZnO film and the substrate lead to extrinsic strain. Therefore, a built-in extrinsic strain is expected to appear owing to the difference of thermal properties between ZnO and Si or *a*-Al_2_O_3_ substrates during growth at T_g_ > RT and after post-growth annealing. The built-in strain causes a shift in the XRD peaks compared with the values observed for single crystalline ZnO, and the strain value depends on the thickness of the film. As the films under study are 100–150 nm thick, significant strain is expected to appear inside the layers.

The strain along the [001] direction (along the *c*-axis) present in the crystallites showing the 002 diffraction peak was calculated using the following expression:

(1) $$\varepsilon_{film}=\left[\frac{d_{001\left(film\right)}-d_{001\left(bulk\right)}}{d_{001\left(bulk\right)}}\right]\cdot 100\%$$

where *d*_001(bulk)_ = 5.205 Å is the distance between (001) planes in ZnO single crystal, while *d*_001(film)_ is the distance between (001) planes calculated from the XRD data. The strain along the [101] direction was calculated analogously with the value *d*_101(bulk)_ = 2.476 Å obtained from the basic formula: $\frac{1}{d^{2}}=\frac{4}{3}\left(\frac{h^{2}+hk+k^{2}}{a^{2}}\right)+\frac{l^{2}}{c^{2}}$ used for the hexagonal lattice (with a = 3.249 Å and c = 5.205 Å) [52].

**Figure 7:**
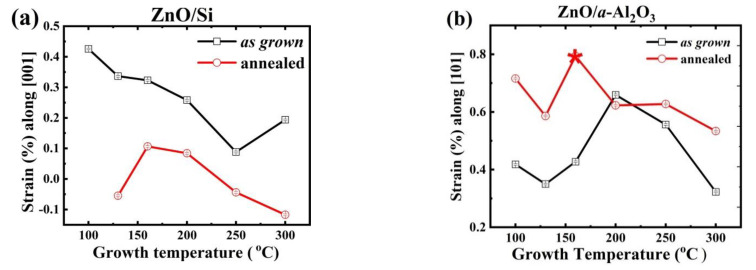
(**a**) The values of strain along the c ([001]) direction in as grown (open square) and annealed(open circle) ZnO/Si films and (**b**) along the [101] direction in as grown (open square) and annealed (open circle) ZnO/a-Al_2_O_3_ films; the star indicates the only layer with the [001] preferred orientation.

Previous investigations [53] on ZnO films using the quartz glass substrate have shown that extrinsic strain generally decreases with increasing T_g_ and can be further relaxed after high temperature annealing or increasing thickness of the film. For the ZnO/Si films investigated here, we found a similar dependence. For as grown ZnO/Si, the strain along the *c*-axis (the [001] direction) was found to be tensile and its magnitude decreased from 0.4 to 0.1% with the rise of T_g_ from 100 to 300 °C. The strain relaxes considerably after annealing, as presented in Figure 7.

For as grown ZnO/*a*-Al_2_O_3_ films, tensile strain was found along the preferred [101] direction. The tensile strain gradually increased with T_g_ from 0.3% to 0.9% at T_g_ = 200 °C and then steadily decreased (see Figure 7b). It is noteworthy that, in contrast to the annealed ZnO/Si(100) films, the strain along the [101] direction increased to a value of 0.5–0.8% after annealing the ZnO/*a*-Al_2_O_3_ films. The crystallites remained preferentially oriented along the [101] direction, and the type of strain along this direction remained tensile. The strain increased strongly at T_g_ = 160 °C when the preferred orientation switched to [001], and it was slightly relaxed for samples grown at T_g_ = 200 °C (see Figure 7b).

It is difficult to find a direct link between the strain in the layers and the dislocations’ density, for both as grown and annealed samples. Annealing is expected to affect both, leading to an increase in crystalline size and a reduction in the grain boundaries. On the other hand, additional strain/dislocations may occur at the interface during the annealing owing to differences in the thermal expansion coefficient between the film and the substrate material.

For ZnO/*a-*Al_2_O_3_ films, both as grown and annealed, the *δ*_avg_ value was much higher than for the ZnO/Si films, and the strain evolution in the case of these two substrates is different. It should be noted that, for ZnO/Si, with the [001] preferred orientation, all crystallites increased considerably after annealing and reached the value of 80–100 nm (Figure 4). In the case of the ZnO/*a*-Al_2_O_3_ films, an increase in the size of the (101) crystallite was observed after RTP, but the crystallite size reached only 30–40 nm. The (001) crystallites were absent or decreased (T_g_ = 160 °C). It might be supposed that the increased strain occurring in the annealed ZnO/*a*-Al_2_O_3_ films inhibited the grain growth in this crystallographic direction. Thus, it can be assumed that the magnitude of strain affected the reduction or increase of crystallite size after annealing.

### 3.2. Connection between XRD and Optical Data

Localised states in semiconductors can be formed by structural defects, impurities, stress/strain, or dislocations. These states might introduce disorder into the electronic structure, leading to a tailing of the band gap. The energy of the band tail is called the Urbach energy (E_u_) [30,54,55] and is defined as follows:

(2) $$\alpha =\alpha_{o\;}exp\left[\left(h\nu \right)/E_{u}\right]$$

where *α* is the absorption coefficient, *α*_o_ is constant, and the Urbach energy characterizes the width of the tail localised states and allows us to estimate the effect of disorder on the bandgap. The optical bandgap and Urbach energy calculated for the as grown and annealed samples deposited on *a*-Al_2_O_3_ were obtained based on the UV transmission spectra (350nm < λ < 450nm) (for more details on the optical data, see [30]). The Urbach was found to change with T_g_ and to be higher for as grown samples than for annealed ones. The latter result could be related to the improvement of the ZnO film quality after annealing, which is also evidenced by the diffraction pattern in Figure 2.

**Figure 8:**
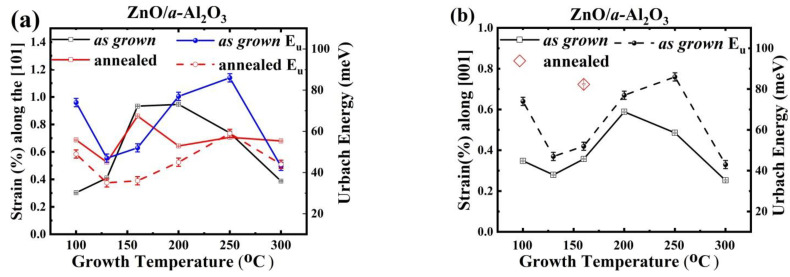
The values of strain (open squares) along (**a**) the [101] direction and (**b**) the [001] direction (along the c-axis) and their correlation with the Urbach energy (solid and open circles) in ZnO/a-Al_2_O_3_ films.

A comparison of the optical and structural properties of the as grown films showed a correlation between the strain present in the films both along both the [101] and the [001] crystallographic orientations with the Urbach energy in the ZnO/a-Al_2_O_3_ films (Figure 8). The annealing process generally reduces the grain boundaries and minimizes the lattice strain, while increasing the crystalline size, which also leads to a lower E_u_ value (Figure 8a). However, in the case of ZnO/*a*-Al_2_O_3_, instead of the expected reduction, an increase in strain was observed after post-growth annealing (Figure 8a). As can be seen in Figure 8, the correlation between E_u_ and strain was weaker after annealing, especially for T_g_ = 160 °C, when switching from the [101] orientation to the [001] orientation occurred.

This means that an increase (decrease) of strain magnitude is accompanied by a corresponding increase (decrease) in the structural disorder in the ZnO/*a*-Al_2_O_3_ films. Based on this, it might be supposed that the developed strain and high concentration of hydrogen impurity creates a subtle perturbation in the density of states near the band edge caused by the electronic structure disorder and affects the exponential dependence of the absorption edge, resulting in an increase of the tail into the band gap (increased Urbach energy (E_u_)) [54,56,57]. This results in a correlation of strain and *δ* value versus E_u_. The opposite behaviour of strain and E_u_ between as grown and annealed ZnO/*a*-Al_2_O_3_ films could again be attributed to a lower concentration of hydrogen or carbon impurities, which dominate the lowering of the Urbach energy and the increase of the optical band gap. For ZnO/*a*-Al_2_O_3_ films, as grown and annealed (see Figure 8), such an interpretation is confirmed.

**Figure 9:**
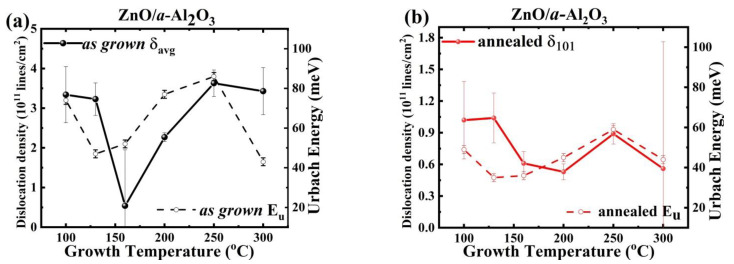
The *δ* value (solid circles) and the Urbach energy (open circles) variation with T_g_ for (**a**) as grown and (**b**) annealed ZnO/a-Al_2_O_3_ films.

Dislocations and grain boundaries can also introduce certain localised states within the gap, and thus influence E_u_ [48]. Indeed, the Urbach energy of as grown ZnO/*a*-Al_2_O_3_ films shows a similar dependence versus T_g_ as the average dislocation density, *δ*_avg_ (Figure 9a). Such a correlation was also found after annealing, but it follows *δ*_101_ rather than the trend of average dislocation density, as shown in Figure 9b. This means that the double δ_001_ value for the sample deposited at 160 °C is not reflected in the Urbach energy (Table 2).

It was observed that E_u_ as well as the optical band gap of ZnO/a-Al_2_O_3_ films grown at the highest temperature (T_g_ ≅ 300 °C) do not change significantly after annealing [58]. It has been reported that the concentration of hydrogen impurity is relatively higher at the lowest ALD growth temperature (100 °C) [21], which at least partially explains the initial high value of strain and E_u_ for films grown at these temperatures. The SIMS results presented in the next paragraph confirm this interpretation.

### 3.3. Electrical Properties and Impurity Concentration

**Figure 10:**
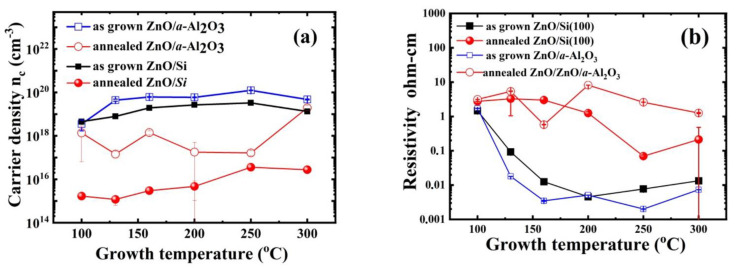
(**a**) Carrier density and (**b**) resistivity versus T_g_ for as grown and annealed ZnO/*a*-Al_2_O_3_ and ZnO/Si(100).

In the case of ALD, there are several options for systematic adjustment of electrical conductivity, as conductivity changes up to three orders of magnitude have been observed in ZnO films only with the variation of T_g_ [21,26,55,59], and this range can be further extended by post-growth annealing. The origin of the conductivity variation is still under debate. It has been tentatively attributed to complexes involving intrinsic defects and hydrogen impurity [21,60,61]. Electrical transport and Hall measurements showed that, in the as grown ZnO/Al_2_O_3_ films (see Table 3), the carrier concentration increased up to 1–2 orders of magnitude in the T_g_ range studied, i.e., from 3.5 × 10^18^ to 1.2 × 10^20^ cm^−3^ (Figure 10a), while the mobility value ranged from 1.2 to 29.2 cm^2^/Vs (see Table 3). Consequently, the resistivity decreased with T_g_ (Figure 10b). In the annealed ZnO/a-Al_2_O_3_ films, the carrier concentration dropped by 1–2 orders of magnitude, but also showed the same behavior, i.e., increases with T_g_. The only exception was the film deposited at 160 °C, where switching to the [001] orientation appeared.

The mobility generally showed lower values after annealing, which was rather unexpected as the crystallite size increased and the *δ*_avg_ value dropped after RTP. These could be related to the higher strain that occurred in ZnO/*a*-Al_2_O_3_ films after annealing.

In the case of the as grown ZnO/Si(100) films, carrier concentration increased from 10^18^ to 10^19^ with a successive T_g_ increase (Figure 10a), while the resistivity decreased from 1 to 10^−3^ Ωcm (Figure 10b). The range of mobility for as grown ZnO/Si films varied from 15 to 31.9 cm^2^/Vs and subsequently increased with T_g_. After annealing, the resistivity increased by 1–2 orders of magnitude and reached values between 0.29 and 8.68 Ωcm. The carrier density measured after the RTP process decreased significantly by 3–4 orders of magnitude to values of 10^15^–10^16^ cm^−3^ compared with the as grown samples. However, these values should be considered with a reservation as the accompanying mobility values were at the level of 200–1000 cm^2^/Vs, which were not reasonable values as they are considerably higher than those reported for single crystal ZnO [62]. It could be suspected that the RTP process carried out at 800 °C, although 3 min short, affects the ZnO/Si interface, leading to the formation of 2D electron gas [63]. Because of this, the mobility values of the annealed ZnO/Si(100) samples are not shown in Table 4.

In summary, the electrical measurements showed that carrier density increased with T_g_ for both substrates and resistivity decreased. Moreover, a significant resistivity drop (1–3 orders of magnitude) was observed after annealing at 800 °C for 3 min for both ZnO/Si as well ZnO/*a*-Al_2_O_3_ films, and resistivity followed the same trend after annealing with respect to T_g_.

Comparison of the level of the carrier concentration with dislocation density for both substrates showed an average dislocation density about three times lower in as grown ZnO/Si films as compared with the as grown ZnO/*a*-Al_2_O_3_ films. It was also accompanied by a higher electron mobility. The only exception appeared at T_g_ = 160 °C, where a change of crystallographic orientation to [001] appeared, and the dislocation density dropped by a few times. This *δ*_avg_ drop might explain a considerable increase in electron mobility observed at this T_g_ for as grown ZnO/*a*-Al_2_O_3_ samples. In the higher T_g_ range (200–300 °C), the correlation between electron mobility and dislocation density was not so clear, and it could be supposed that other effects, as impurities, also influenced electron mobility. On the other hand, it can be expected that the value of dislocation density, *δ*_avg_, which for the polycrystalline films can be treated as a parameter describing the amount of structural defects related to grain boundaries, could be associated with higher electron concentration, as defects and defect complexes are easily bound to grain boundaries. Indeed, the higher electron concentration observed in the ZnO/*a*-Al_2_O_3_ layers compared with the ZnO/Si(100) films was accompanied by a higher dislocation density in these films (see Table 1, Table 2, Table 3 and Table 4). After annealing, when crystallite sizes increased and *δ*_avg_ decreased, the carrier concentration was also lower in ZnO/*a*-Al_2_O_3_ layers.

Impurity investigations are necessary to gain a deeper insight into the problem of conductivity differences. Monitoring of hydrogen impurity is unavoidable, because both precursors used (DEZn, H_2_O) contain hydrogen, which can directly, as an interstitial hydrogen, H_i_, or indirectly, as part of native-point-defect-hydrogen-impurity complexes, influence the electrical conductivity of the ZnO layers [2,21].

**Figure 11:**
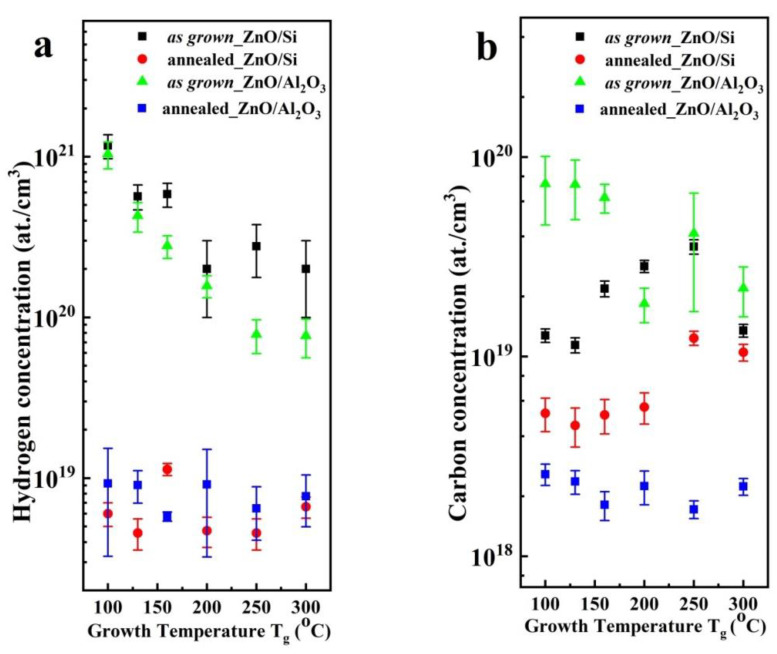
Hydrogen (**a**) and carbon (**b**) concentration in as grown (black squares) and annealed (red squares) ZnO/Si films and as grown (green triangle) and annealed (blue squares) ZnO/a-Al_2_O_3_ grown at different T_g_.

SIMS measurements showed that hydrogen concentration in both the as grown ZnO/Si and ZnO/*a*-Al_2_O_3_ films was 10^21^ cm^−3^ (higher for T_g_ = 100 °C) and decreased by about two orders of magnitude (10^19^–10^18^ cm^−3^) after annealing (Figure 11a), which likely accounts for a decrease in carrier concentration by two or more orders of magnitude after annealing. Similar results have been reported for thick ZnO films [21], where we also observed a correlation between electron and hydrogen concentration. However, it should be stressed that, in each case, the hydrogen concentration was higher than the electron density, indicating that at least some of the hydrogen does not play the role of a donor.

In our recent work [64], it has already been demonstrated that the contribution of hydrogen deriving from the oxygen precursor (H_2_O) can be completely removed by rapid thermal annealing, while hydrogen deriving from the DEZn precursor is more robust. It should be noted that, in the annealed ZnO/Si samples, the concentrations of hydrogen and carbon were almost at the same level. It can be predicted that the hydrogen remaining after annealing is in the form of hydrocarbon groups, which means that DEZn can be the main source of hydrogen in the annealed ALD-ZnO thin films [64].

It is worth noting that, for thin ZnO/*a-*Al_2_O_3_ films grown at extreme T_g_ (100 and 300 °C), although the average crystallite size increased and hydrogen concentration decreased considerably after post-growth annealing, the carrier concentration remained at a level comparable to that measured for as grown films.

The concentration of carbon was found to be one order of magnitude lower than the concentration of hydrogen (Figure 11a,b). It was 1–3 × 10^19^ cm^−3^ for the ZnO/Si(100) layers and about 2–3 times higher (2–9 × 10^19^ cm^−3^) for the ZnO/*a*-Al_2_O_3_ layers (Figure 11b). After annealing, the carbon concentration decreases in both films, but this decrease is more significant in the ZnO/*a*-Al_2_O_3_ layers than in the ZnO/Si films and, consequently, carbon concentration is lower in the former layers. It should be noted that, at higher T_g_ (200–300 °C), the carbon concentration is almost the same in all as grown films. Nevertheless, annealing affected the carbon concentration in the ZnO/Si films much less than the ZnO/*a*-Al_2_O_3_ films, for which two orders of magnitude lower carbon concentration was observed after annealing.

The nitrogen concentration in both films types was in the range of 10^17^–10^18^ atom/cm^−3^ and does not change after annealing (not shown here).

### 3.4. Grain Boundary Effect on Electrical Conductivity

The main mechanism for describing electrical conductivity (δ) in polycrystalline films is based on the grain boundary (GB) scattering model [65,66]. Grain boundaries (GBs) exist in a polycrystalline film at the interfaces of the crystallites and may play a crucial role in determining the conductivity of the polycrystalline film. The potential energy barriers exist around the GBs as a result of band bending induced via majority carriers (e.g., electrons) trapped at surface states. The GB model states that a decrease in crystallite size causes an increase in GB scattering, resulting in a decrease in the electrical conductivity. In this model, the variation in electrical conductivity with temperature is strongly determined by whether the grains are completely or partially depleted of charge carriers. When the grains are only partially depleted, the charge carrier distribution is strongly inhomogeneous because the depletion layer barriers are adjacent to grain boundaries. In this regime, the Hall coefficient (R_H_) and the concentration are not connected by the simpler relation, i.e., R_H_ ∝ 1/n_c_, which is no longer valid. Under this regime, the GB model yields the following expression for electrical conductivity $\sigma =\left(\frac{De^{2}n}{2m^{*}k_{B}T}\right)\;exp\left[-\left(\;\frac{E_{b}}{k_{B}T}\;\right)\;\right];$ where e is the charge on electron, D is the average crystallites’ size, $E_{b}$ is the energy barrier at the grain boundary given by $E_{b}=E_{C}-E_{F}+e\phi =\frac{D^{2}e^{2}N_{d}}{8}$, k_B_ is the Boltzmann constant, and m* is the effective mass of charge carriers, while *E*_C_—energy of conduction band minimum, *E*_F_—Fermi level, ϕ—grain boundary potential barrier, *N*_d_—donor concentration, and *ε*—low frequency dielectric constant (for ZnO ≈ 8.5).

The GB effect on electrical parameters can be estimated by comparing the Debye length (*L*_D_) with the average grain size (D). The Debye length is the length scale over which the local electric field affects the distribution of free charge carriers in a semiconductor. It decreases with increasing concentration of free charge carriers and its value can be estimated [59] as $L_{D}={\left(\frac{k_{B}T_{0}}{N_{d}e^{2}}\right)}^{1/2}$, where *ε*_0_ is the permittivity of the vacuum. It can be safely assumed that the donor concentration is *N*_d_ as undoped ZnO is a heavily *n*-type material.

The approximate value of *L*_D_ and half grain size (D/2) for as grown and annealed samples deposited at different T_g_ is presented in Table 5. In the regime where D/2 > *L*_D_, the interfacial trap states create potential barriers in the GB regions. In these polycrystalline films, a large number of localized defect states can be expected near the grain boundaries, acting as scattering centres for charge carriers.

When L_D_ is greater than D/2, the potential barriers are not present and the conduction band becomes flat, resulting in a constant carrier concentration (*n*) across the grain, and GB scattering is not dominant.

A comparison of the Debye length with carrier concentration and the grain sizes (Table 5) shows that all as grown films do not satisfy the L_D_ > D/2 condition. This implies that, in all as grown films under study, the charge carriers experience the grain boundary potential barriers.

However, after annealing, at the low temperature range (T_g_ 100–160 °C), the condition L_D_ > D/2 is fulfilled for the ZnO/Si(100) samples, while it is not satisfied for ZnO/a-Al_2_O_3_ samples as well as for ZnO/Si(100) deposited at the high T_g_ range (200–300 °C). This means that, in the annealed ZnO/Si(100) films grown below 160 °C, GB scattering is probably not dominant. Higher dislocation density observed for the ZnO/Si films grown at T_g_ >160 °C a (Table 1) can be attributed to the switching phenomenon. The increased dislocation density probably influenced the carrier concentration in these films, which is higher. Therefore, we may speculate that the switching phenomena occurring at T_g_ of 160 °C alter the scattering mechanism in the ZnO/Si (100) films.

For all as grown and annealed ZnO/a-Al_2_O_3_ films, the L_D_ > D/2 condition is not fulfilled in the whole range of T_g_. This implies that the effect of the GB potential barrier on conductivity should always be considered. These results differ from previous reports on 900 nm thick ZnO/Al_2_O_3_ films grown by ALD, where the L_D_ > D/2 condition was satisfied for the films deposited below 200 °C. It can be concluded that the proximity of the interface has a significant effect on the charge carrier scattering, which is interfacial and annealing dependent. Moreover, the homogeneity of the charge carrier distribution is different for annealed ZnO/Si and ZnO/Al_2_O_3_ layers. A deeper insight into the problem of carrier scattering requires a detailed investigation of the carrier mobility as a function of temperature, which is beyond the scope of the present study.

It should be noted that the above analysis is only approximate because it is based on the crystallite size calculated from diffractograms shown in Figure 1 and Figure 2, so the size of the crystallites perpendicular to the growth directions was examined in the considerations of the scattering mechanism.

## 4. Summary and Outlook

In summary, it was shown that, despite crystallographic mismatch, the dominant orientation and quality of the 100 nm thick polycrystalline ZnO films grown on Si(100) and *a*-Al_2_O_3_ are different. As for the layers deposited at the same temperature, the films deposited on silicon showed reduced strain and dislocation density compared with the films deposited on sapphire. The three-minute annealing in oxygen at 800 °C significantly improved the quality of all ZnO layers, as evidenced by lower dislocation density as well as reduced hydrogen and carbon impurities.

Tensile strain was observed for as grown ZnO/Si(100) and ZnO/*a*-Al_2_O_3_ films, higher for the latter, but its evolution after annealing was different. The strain was reduced in the ZnO/Si(100) films, while it increased in the ZnO/*a*-Al_2_O_3_ layers after a short annealing at 800 °C. For the latter films, a good correlation was found between the degree of strain, Urbach energy, and dislocation density as a function of T_g_. As expected, E_u_ was reduced after annealing.

Comparing the films deposited at different T_g_, the films deposited at lower temperatures, 100–130 °C, showed a lower carrier concentration, which was accompanied by a high hydrogen content. The Debye length, L_D_, was less than half the crystallite size, D/2, for all as grown samples and annealed ZnO/*a*-Al_2_O_3_ films, according to the grain boundary model. Thus, the grains were only partially depleted and the charge carrier distribution was highly inhomogeneous in these films, so the effect of GB potential barrier on conductivity should be taken into account. On the other hand, annealed ZnO/Si(100) samples deposited at temperatures below 200 °C satisfied the L_D_ > D/2 condition, implying that the grains are fully depleted and the charge carriers might be assumed to be transported without experiencing GB scattering.

The uniform carrier distribution envisaged for annealed ZnO/Si(100) films deposited at T_g_ of 160 °C or below (i.e., O-rich), as well as lower dislocation density and strain, predesignated these films for electronic applications, such as field effect transistors or memory devices. In turn, the ZnO films deposited at 200 °C are predestined for transparent conductive oxide applications, as they combine high conductivity with surface smoothness at the atomic scale.

In conclusion, it was shown that the type of substrate affects dislocation density, strain, and electrical transport in polycrystalline ZnO films, and thus the conductivity of the film. According to our results, the choice of an Si substrate (which is also industry friendly) seems to be better in this sense. The presented studies fit into the current discussion on native point defect complexes by showing that not only the hydrogen content (similar in both series studied) and/or the growth conditions (O-rich or Zn-rich) determine the conductivity of the material. The level of structural defects also plays an important role, indirectly pointing to their possible role in the formation of hydrogen impurity–native point defect complexes providing shallow defect levels.

## Figures and Tables

**Table 1:** Dislocation density (δ) for as grown and annealed ZnO/Si (100) films calculated based on the XRD data.

T_g_ (°C)	δ_(002)_ (10^11^ lines/cm^2^)	δ_(100)_ (10^11^ lines/cm^2^)	δ_avg_ (10^11^ lines/cm^2^)	δ_(002)_ (10^11^ lines/cm^2^)	δ_(100)_ (10^11^ lines/cm^2^)	δ_avg_ (10^11^ lines/cm^2^)
	ZnO/Si As Grown			ZnO/Si Annealed		
100	2.05	1.22	1.62	-	0.11	0.11
130	1.61	1.16	1.38	0.11	0.10	0.10
160	1.72	0.98	1.43	0.17	0.10	0.14
200	4.70	1.49	3.51	3.37	0.10	0.46
250	0.74	-	0.74	0.14	-	0.14
300	1.13	-	1.13	0.20	-	0.20

**Table 2:** Dislocation density (*δ*) for as grown and annealed ZnO/*a*-Al_2_O_3_ films calculated based on the XRD data.

T_g_ (°C)	δ_(101)_ (10^11^ lines/cm^2^)	δ_(002)_ (10^11^ lines/cm^2^)	δ_avg_ (10^11^ lines/cm^2^)	δ_(101)_ (10^11^ lines/cm^2^)	δ_(002)_ (10^11^ lines/cm^2^)	δ_avg_ (10^11^ lines/cm^2^)
	ZnO/*a*-Al_2_O_3_ As Grown			ZnO/*a*-Al_2_O_3_ Annealed		
100	5.49	1.25	3.34	1.02	-	1.02
130	4.33	1.10	3.23	1.04	-	1.04
160	5.12	0.45	0.54	0.61	1.33	1.22
200	2.43	1.27	2.27	0.53	-	0.53
250	3.85	2.58	3.63	0.89	-	0.89
300	4.39	1.90	3.43	0.56	-	0.56

**Table 3:** Electrical parameters and thickness of as grown and annealed ALD- ZnO/*a*-Al_2_O_3_ thin films.

T_g_ (°C)	As Grown Samples Carrier Density n_c_ (cm^−3^)/Mobility(cm^2^/Vs) ZnO/*a*-Al_2_O_3_ n_c_(cm^−3^)/µ (cm^2^/Vs) ρ(Ωcm)		Annealed Samples Carrier Density n_c_ (cm^−3^)/Mobility(cm^2^/Vs) ZnO/*a*-Al_2_O_3_ n_c_(cm^−3^)/µ (cm^2^/Vs) ρ(Ωcm)	
100	3.5 × 10^18^/1.2	1.7	1.4 × 10^18^/3.7	3.15
130	4.4 × 10^19^/8.3	1.8 × 10^−2^	1.4 × 10^17^/10.0	5.38
160	6.2 × 10^19^/29.2	3.5 × 10^−3^	1.4 × 10^18^/8.8	0.58
200	5.9 × 10^19^/20.6	5.1 × 10^−3^	1.8 × 10^17^/10.0	8.14
250	1.2 × 10^20^/25.1	2.0 × 10^−3^	1.6 × 10^17^/19.5	2.60
300	4.7 × 10^19^/25.1	7.3 × 10^−3^	2.0 × 10^19^/8.0	1.25

**Table 4:** Electrical parameters and thickness of as grown and annealed ALD- ZnO/Si(100) thin films (mobility of annealed samples is not included, see explanation in the text, *p*. 14, lines 506–508).

T_g_ (°C)	As Grown Samples Carrier Density n_c_ (cm^−3^)/Mobility(cm^2^/Vs) ZnO/Si(100) n_c_(cm^−3^)/µ (cm^2^/Vs) ρ(Ωcm)		Annealed Samples Carrier Density n_c_ (cm^−3^) & Resistivity ρ(Ωcm), ZnO/Si(100) n_c_(cm^−3^) ρ(Ωcm)	
100	4.5 × 10^18^/17.3	8.4 × 10^−2^	1.7 × 10^15^	3.12
130	7.9 × 10^18^/15.2	5.2 × 10^−2^	1.2 × 10^15^	3.52
160	1.9 × 10^19^/22.5	1.4 × 10^−2^	3.0 × 10^15^	2.96
200	2.7 × 10^19^/22.6	1.0 × 10^−2^	4.7 × 10^15^	8.68
250	3.3 × 10^19^/22.4	8.5 × 10^−3^	3.6 × 10^16^	2.9 × 10^−1^
300	1.3 × 10^19^/31.9	1.5 × 10^−2^	2.7 × 10^16^	7.6 × 10^−1^

**Table 5:** Approximate values of the Debye length and a half of the crystallite size for different carrier concentration for as grown and annealed samples of thin ZnO films on *a*-Al_2_O_3_ (top) and Si (bottom).

**ZnO/*a*-Al_2_O_3_**								
**As Grown ZnO/*a*-Al_2_O_3_**					**Annealed ZnO/*a*-Al_2_O_3_**			
**Tg (°C)**	**Hall Conc. (cm^−3^)**	$L_{D}\;\left(nm\right)$	**Half of Crystallite Size, D/2 (nm)**		**Hall Conc. (cm^−3^)**	***L_D_ (nm)***	**Half of Crystallite Size, D/2 (nm)**	
			**[101]**	**[002]**			**[101]**	**[002]**
100	3.50 × 10^18^	1.86	6.75	14.15	1.36 × 10^18^	2.98	15.7	NA
130	4.40 × 10^19^	0.52	7.6	15.08	1.44 × 10^17^	9.18	15.49	NA
160	6.15 × 10^19^	0.44	6.99	23.64	1.41 × 10^18^	2.93	20.18	13.72
200	5.93 × 10^19^	0.45	10.15	14.06	1.78 × 10^17^	8.26	21.73	NA
250	1.24 × 10^20^	0.05	8.05	9.85	1.64 × 10^17^	8.61	16.78	NA
300	4.73 × 10^19^	0.51	7.55	11.49	1.95 × 10^19^	0.79	21.06	NA
**ZnO/Si(100)**								
**As Grown ZnO/Si(100)**					**Annealed ZnO/Si(100)**			
**T_g_ (°C)**	**Hall Conc.** **(cm^−3^)**	***L_D_*** **(nm)**	**Half of Crystallite Size D/2 (nm)**		**Hall Conc.** **(cm^−3^)**	***L_D_*** **(nm)**	**Half of Crystallite Size D/2 (nm)**	
			**[100]**	**[002]**			**[100]**	**[002]**
100	4.53 × 10^18^	1.64	14.3	11.0	1.67 × 10^15^	85.16	46.8	NA
130	7.87 × 10^18^	1.24	14.7	12.5	1.18 × 10^15^	101.31	49.4	47.9
160	1.93 × 10^19^	0.79	16	12.1	2.99 × 10^15^	63.64	49.1	38.1
200	2.66 × 10^19^	0.68	12.9	7.3	4.71 × 10^15^	50.71	51.1	NA
250	3.29 × 10^19^	0.61	NA	18.4	3.57 × 10^16^	18.42	NA	43.0
300	1.33 × 10^19^	0.95	NA	14.9	2.74 × 10^16^	21.02	NA	35.4

## Data Availability

Data is contained within the article are available in Supplementary Material.

## Supplementary Materials

The following are available online at https://www.mdpi.com/article/10.3390/ma14144048/s1, Figure S1: XRD analysis of as grown (a–f) and annealed (g–l) ZnO/Si, Figure S2: XRD analysis of as grown (a–f) and annealed (g–l) ZnO/a-Al_2_O_3_.
