# Supplementary material for: Structural Properties of Thin ZnO Films Deposited by ALD under O-Rich and Zn-Rich Growth Conditions and Their Relationship with Electrical Parameters
Source: Materials (Basel). 2021 Jul 20;14(14):4048. doi: 10.3390/ma14144048 (PMC8307850; doi:10.3390/ma14144048)

Sushma Mishra et al., Supplementary Material

Figure S1. XRD analysis of as grown (a-f) and annealed (g-l) ZnO/Si

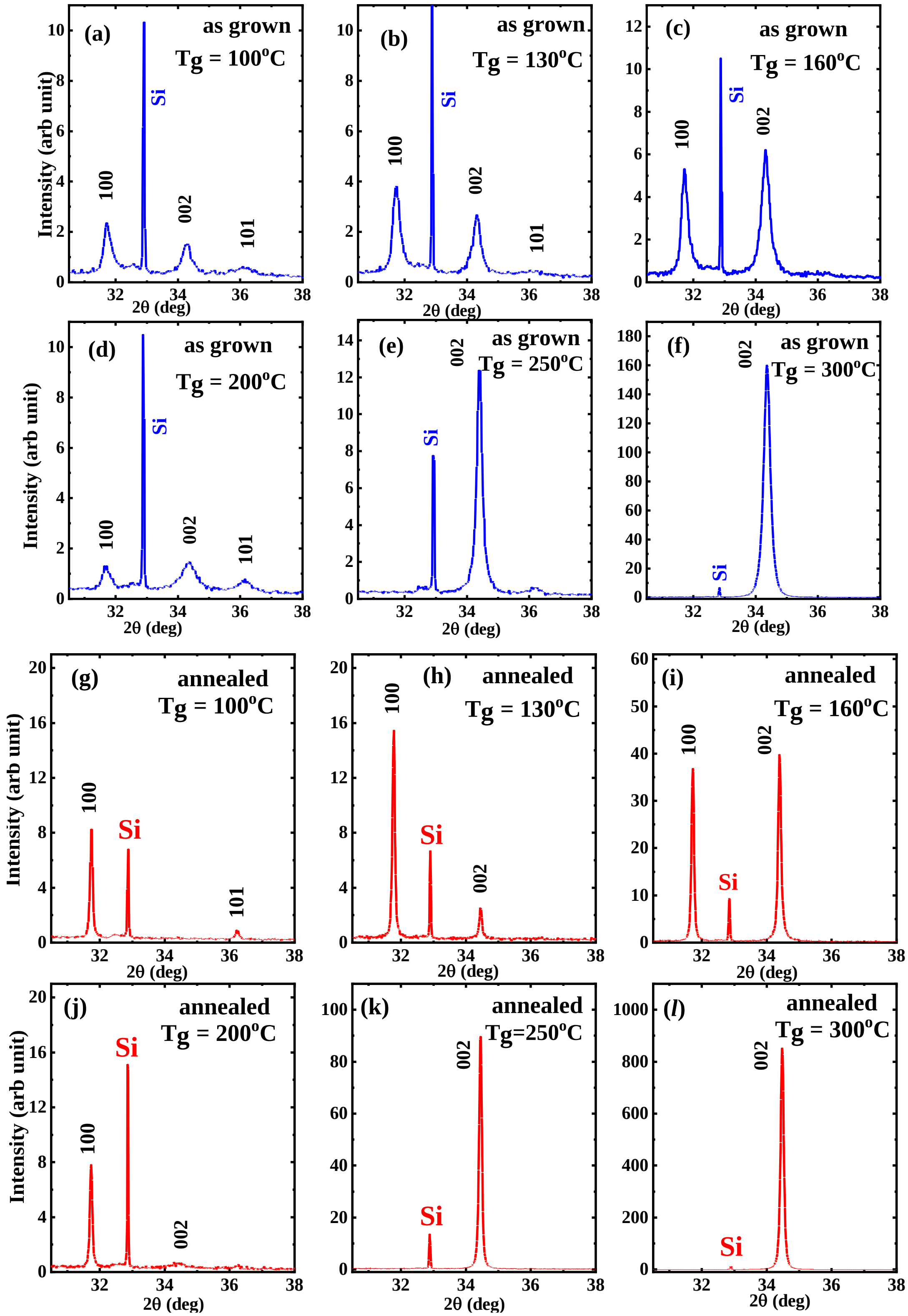

Figure S2. XRD analysis of as grown (a-f) and annealed (g-l) ZnO/*a*-Al_2O_3

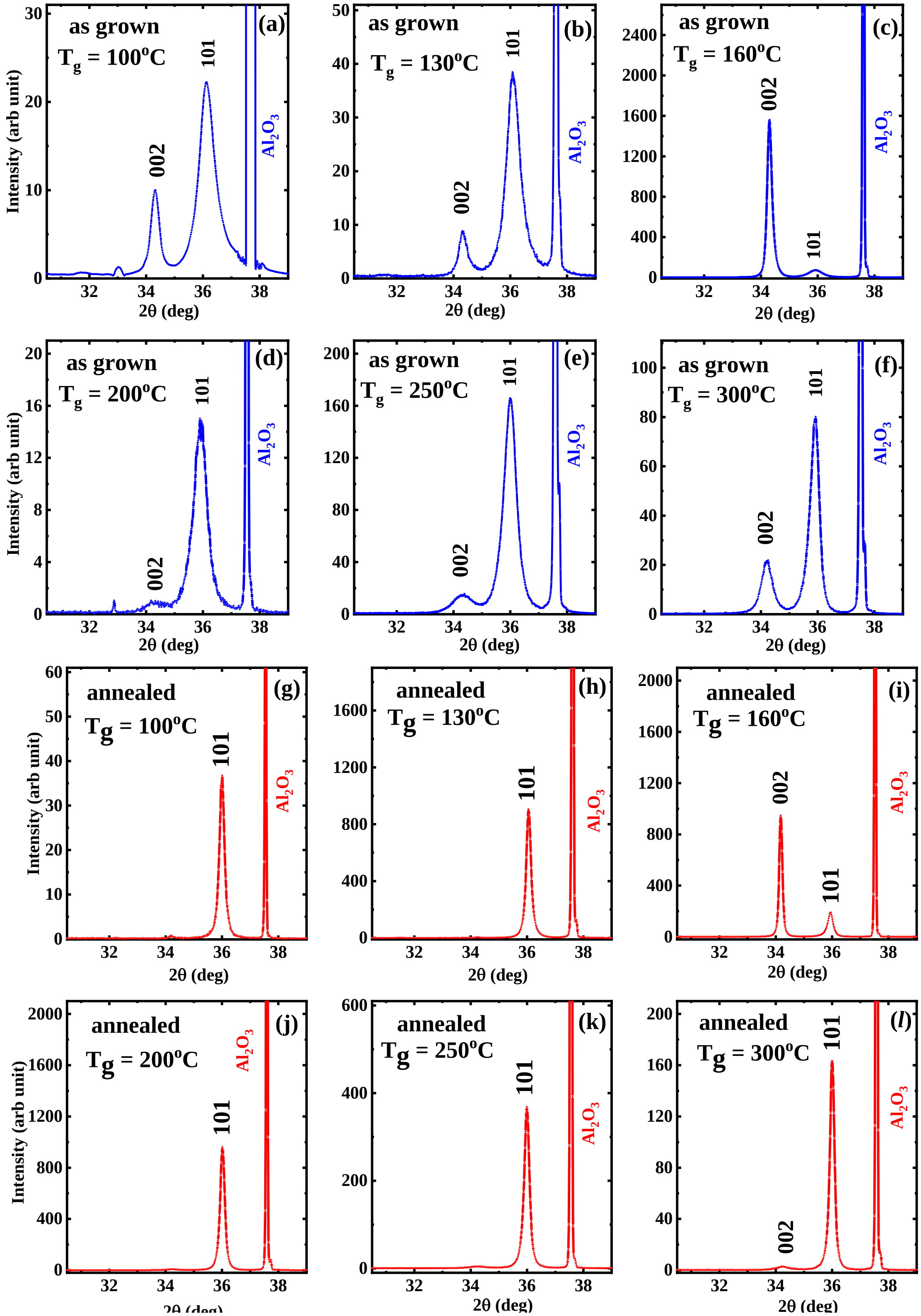

Supplement: Supplementary file 1 [file materials-14-04048-s001.zip › materials-1258290-supplementary.pdf]
